# Effect of pH and Salinity on the Ability of *Salmonella* Serotypes to Form Biofilm

**DOI:** 10.3389/fmicb.2022.821679

**Published:** 2022-04-07

**Authors:** Sara Petrin, Marzia Mancin, Carmen Losasso, Silvia Deotto, John Elmerdahl Olsen, Lisa Barco

**Affiliations:** ^1^Microbial Ecology and Microorganisms Genomics Laboratory - SCS1, Istituto Zooprofilattico Sperimentale delle Venezie, Legnaro, Italy; ^2^Department of Veterinary and Animal Sciences, Faculty of Health and Medical Sciences, University of Copenhagen, Frederiksberg, Denmark; ^3^OIE and National Reference Laboratory for Salmonellosis, Istituto Zooprofilattico Sperimentale delle Venezie, Legnaro, Italy; ^4^Clinical Diagnostics Laboratory - SCT4, Istituto Zooprofilattico Sperimentale delle Venezie, Basaldella di Campoformido, Italy

**Keywords:** *Salmonella*, biofilm, pH, salinity, *S*. Dublin

## Abstract

*Salmonella* is a major cause of food-borne infections in Europe, and the majority of human infections are caused by only a few serotypes, among them are *Salmonella enterica* subsp. *enterica* serotype Enteritidis (hereafter *Salmonella* Enteritidis), *Salmonella* Typhimurium, and the monophasic variant of *S*. Typhimurium. The reason for this is not fully understood, but could include virulence factors as well as increased ability to transfer *via* the external environment. Formation of biofilm is considered an adaptation strategy used by bacteria to overcome environmental stresses. In order to assess the capability of different *Salmonella* serotypes to produce biofilm and establish whether this is affected by pH and salinity, 88 *Salmonella* isolates collected from animal, food, and human sources and belonging to 15 serotypes, including those most frequently responsible for human infections, were tested. Strains were grown in tryptic soy broth (TSB), TSB with 4% NaCl pH 4.5, TSB with 10% NaCl pH 4.5, TSB with 4% NaCl pH 7, or TSB with 10% NaCl pH 7, and biofilm production was assessed after 24 h at 37°C using crystal violet staining. A linear mixed effect model was applied to compare results from the different experimental conditions. Among the tested serotypes, *S*. Dublin showed the greatest ability to form biofilm even at pH 4.5, which inhibited biofilm production in the other tested serotypes. *Salmonella* Senftenberg and the monophasic variant of *S*. Typhimurium showed the highest biofilm production in TSB with 10% NaCl pH 7. In general, pH had a high influence on the ability to form biofilm, and most of the tested strains were not able to produce biofilm at pH 4.5. In contrast, salinity only had a limited influence on biofilm production. In general, serotypes causing the highest number of human infections showed a limited ability to produce biofilm in the tested conditions, indicating that biofilm formation is not a crucial factor in the success of these clones.

## Introduction

*Salmonella* are Gram-negative bacteria that cause illnesses in humans, ranging from self-limiting gastroenteritis to severe fever and bacteremia in both developed and developing countries (Bell and Kyriakides, 2009). According to the latest EFSA report on zoonosis, a total of 87,923 confirmed cases of salmonellosis were reported in 2019 in the European Union, thus confirming *Salmonella* spp. as the second most commonly reported zoonoses (EFSA and ECDC, 2021a). More than 2,600 *Salmonella* serotypes have been identified (Grimont and Weill, 2007); however, 74% of the 87,923 confirmed human cases are caused by just five serotypes, namely, *Salmonella enterica* subsp. enterica serotype Enteritidis (*Salmonella* Enteritidis), *Salmonella* Typhimurium, and its monophasic variant, *Salmonella* Infantis and *Salmonella* Newport (EFSA and ECDC, 2021a). The reason why these serotypes are so commonly isolated from human infections is not fully understood, but can include both virulence factors and factors that enable them to persist in and transfer through the food chain more efficiently than other serotypes. Poultry meats and eggs are among the primary food vehicles causing salmonellosis foodborne outbreaks (EFSA and ECDC, 2021a; Guillier et al., 2021); thus, control measures are implemented in poultry sector and current strategies in Europe focus on specific serotypes. For example, the serotypes mentioned above, with the exception of *S*. Newport, are targets for *Salmonella* control in poultry populations, according to the EU Regulation (EC) 2160/2003. So are other serotypes, such as *Salmonella* Hadar and *Salmonella* Virchow although these serotypes are rarely isolated from human salmonellosis, with only 469 reported cases for *S*. Virchow and 297 reported cases for *S*. Hadar in 2019 in the EU (ECDC, n.d.). Based on epidemiological evidences collected in Italy, Leati et al. (2021) suggested that other serotypes, such as *Salmonella* Derby and *Salmonella* Napoli, should be considered for control at primary production level, due to their frequent isolation in human infections. In addition, it has increasingly been reported that specific clones of different serotypes, rather than the serotype as such, are responsible for emergent spread in both humans and the food chain (Coipan et al., 2020; García-Soto et al., 2020; Mastrorilli et al., 2020; EFSA and ECDC, 2021b). Often, these clones are characterized by the acquisition of large conjugative plasmids providing resistance features and virulence-associated properties (Franco et al., 2015; Alba et al., 2020; García-Soto et al., 2020). Thus, the current control measures, relying upon seroype-linked strategies, could miss highly pathogenic *Salmonella* strains as well as call for action even though the serotype involved seems to have low ability to either transfer to or cause infection in humans.

Biofilms, defined as consortiums of multiple bacterial cells, embedded in a self-produced extracellular polymeric matrix and attached to a surface (Hall-Stoodley et al., 2004; Bjarnsholt, 2013), have both been considered to enhance virulence of *Salmonella* spp. (Borges et al., 2018; Moraes et al., 2018) and to increase persistence in nonhost environments, especially food processing environments (Steenackers et al., 2012). The ability of *Salmonella* strains to form biofilm is considered a key strategy for their survival, and it has been linked to an increased occurrence in outbreaks (Steenackers et al., 2012; O’Leary et al., 2015; Moraes et al., 2018) and to increased tolerance to antimicrobials, disinfectants, and other environmental stresses (Bridier et al., 2011; Esbelin et al., 2018; Cadena et al., 2019; Tassinari et al., 2019). Similarly to other microbial behaviors, the ability to form biofilm is influenced by intrinsic and extrinsic factors, including temperature, pH, water activity (a_w_), and nutrient availability (Alvarez-Ordóñez et al., 2019; Lianou et al., 2020), but also microbial species, serotypes, and lineages (Díez-García et al., 2012; MacKenzie et al., 2017; Lee et al., 2019). Studies have attempted to correlate biofilm phenotype to *Salmonella* serotype and environmental persistence, but results were conflicting (MacKenzie et al., 2017), especially when strain variability within serotypes was taken into account (Lianou and Koutsoumanis, 2012; Lee et al., 2019), and currently it is unknown whether the serotypes frequently associated with human infections differ from other *Salmonella* serotypes in their ability to produce biofilms. Thus, the main objective of the present study was to compare the biofilm production capability of *Salmonella* serotypes isolated with different frequencies from human infections and assess whether biofilm formation was affected by osmolarity (NaCl concentration) and pH.

## Materials and Methods

### Strain Selection

A total of 88 *Salmonella* isolates, maintained at the Istituto Zooprofilattico Sperimentale delle Venezie, were selected to study their ability to form biofilm. The strains belonged to 15 different serotypes, namely, *Salmonella* Derby, *Salmonella* Dublin, *Salmonella* Enteritidis, *Salmonella* Hadar, *Salmonella* Infantis, *Salmonella* Kentucky, *Salmonella* Livingstone, *Salmonella* Mbandaka, *Salmonella* Montevideo, monophasic variant of *S*. Typhimurium (MVST), *S*. Newport, *Salmonella* Rissen, *Salmonella* Senftenberg, *Salmonella* Thompson, and *S*. Typhimurium. For each serotype, two strains were included from each of the sources animals, food, and humans, with the exception of *S*. Dublin and *S*. Mbandaka, for which only one human isolate was retrieved. Therefore, six strains were tested for each serotype, except *S*. Dublin and *S*. Mbandaka. A detailed description of isolates is reported in Supplementary Table S1. The strains were stored at −80°C in cryobank tubes with preservative medium (Copan Diagnostics, CA, United States) and were tested for purity before use. For inoculation, each strain was transferred from the stock cultures into tryptic soy broth (TSB) and incubated overnight at 37°C. The grown cultures were used for inoculation into different media for subsequent quantification of biofilm production.

### Experimental Conditions and Biofilm Assay

The ability of the selected strains to form biofilm was measured in TSB without and with 4 and 10% w/v NaCl and at pH values 4.5 and 7. The NaCl concentrations corresponded to water activity (a_w_) of 0.976 and below 0.959 (Lianou and Koutsoumanis, 2012). *In vitro* evaluation of biofilm production was performed according to Stepanović et al. (2007), using a colorimetric microtiter plate method that measures the optical density of biofilm mass after staining with crystal violet. Specifically, overnight cultures grown at 37°C in TSB and subsequently diluted by adding TSB to a final OD_600_ of 0.2 (corresponding to approximately 1 × 10^8^ CFU/ml). Around 20 μl of each isolate was inoculated in 180 μl of broth in microtiter plates with flat bottom (Greiner Bio-One). For each isolate, six replicates were inoculated in every tested condition, and 12 negative controls, i.e., uninoculated broth, were included in each plate. The plates were incubated for 24 h at 37°C. After the incubation period, the content of the wells was discard by means of a vacuum pump and washed twice with 200 μl of distilled water to remove nonadherent bacterial cells. The adherent cells were fixed with 200 μl of methanol (VWR Chemicals) in each well for 20 min. The microtiter plates were then emptied and air-dried for at least 1 h at room temperature, and 200 μl of 2% crystal violet (DELCON) was added per well to stain the biofilm mass for 15 min. Stain in excess was removed by filling the plates under tap water and empting by inversion; after tapping on adsorbent paper, plates were dried under the hood overnight. To solubilize the crystal violet bound to the biofilm, 200 μl of absolute ethanol (Carlo Erba Reagents) was added in each well. Plates were kept in agitation (180 rpm) for 15 min at room temperature before reading on a Tecan Sunrise (Tecan) spectrophotometer. Optical density (OD) was read at 570 nm.

### Statistical Analysis

Two approaches were used to analyze the OD measurements: a qualitative classification of the biofilm production and a quantitative analysis of the observed OD values.

In order to classify the isolates according to their ability to form biofilm, the average OD (OD_a_) of the six replicates was calculated for each tested strain and the negative controls. A cutoff OD (OD_c_) was defined as three SDs above the OD_a_ of the negative control. Based on the OD produced by bacterial biofilms, strains were classified as “no biofilm producers”: OD_a_ ≤ OD_c_; “weak biofilm producers”: OD_c_ < OD_a_ ≤ 2OD_c_; “moderate biofilm producers”: 2OD_c_ < OD_a_ ≤ 4OD_c_; and “strong biofilm producers”: 4OD_c_ < OD_a_ (Stepanović et al., 2000). Descriptive statistical analysis was performed to summarize the OD data of each serotype in the different experimental conditions. Box plots were used to synthesize the data, providing the principal measures of central tendency and dispersion. To verify whether significant differences existed between the average OD values of the selected serotypes in the different experimental conditions, a linear mixed model was applied. The model takes into account the experimental design, considering a random effect for the nested replicates of each serotype tested in every condition. For this analysis, the OD_a_ of negative controls calculated in each plate was subtracted from the OD of each isolate well in the same plate, in order to make the results between serotypes and among different experimental conditions comparable. The variables “serotypes,” “experimental condition,” and their interaction were included in the model as fixed factors. Further details about the model are available as Supplementary Material and Method.

To evaluate the significance of the overall effect of fixed factors specified in the model, Type III F test was applied. For each fixed factor of the mixed models, *post hoc* pairwise comparisons were performed to further clarify those differences. In the case of multiple tests, the Tukey adjusted *p*-values were provided. Values of *p* < 0.05 were considered significant. SAS 9.4 software was used to perform the analysis (Cibin et al., 2017; Roccato et al., 2018).

## Results

Regardless of the serotype, the majority of *Salmonella* isolates tested in TSB felt into the “weak biofilm producer” category (*n* = 61), some were “moderate biofilm producer” (*n* = 22), only one isolate was categorized as “no biofilm producer,” and four as “strong biofilm producers.” When salt was added to TSB, a greater number of isolates felt into the “moderate producer category” (*n* = 47 and *n* = 60 for 4 and 10% NaCl, respectively). Under these conditions, also the number of “strong biofilm producers” stains increased (*n* = 5 and *n* = 10 isolates for 4 and 10% NaCl, respectively), while no isolate was categorized as no biofilm producer (Table 1).

**Table 1:** Frequency of *Salmonella* isolates (*n* = 88) per categories: “no biofilm producer,” “weak producer,” “moderate producer,” and “strong producer” when tested in different conditions.

	TSB	TSB pH 74% NaCl	TSB pH 710% NaCl	TSB pH 4.54% NaCl	TSB pH 4.510% NaCl
No producer	1	0	0	11	2
Weak producer	61	36	18	72	80
Moderate producer	22	47	60	3	4
Strong producer	4	5	10	2	2

Categories are defined as reported above, according to Stepanović et al. (2000).

Looking in detail at the changes in the stains’ ability to form biofilm (Supplementary Table S2), it is possible to note that 38 isolates showed a change in their ability to form biofilm in the experimental conditions at pH 7, while 14 isolates showed a change in their ability to form biofilm in the experimental conditions at pH 4.5. Moreover, in the experimental conditions at pH 7, a number of 30 stains (79%) showed an increase in the ability to form biofilm when exposed to 10% NaCl, and 12 strains (86%) showed an increase in the ability to form biofilm when exposed to 10% NaCl in the experimental conditions at pH 4.5.

No major differences were observed in biofilm forming ability depending on the source of isolation, as 94.7, 96.7, and 99.3% of strains isolated from animals, food, and humans were categorized as biofilm producers.

**Figure 1:**
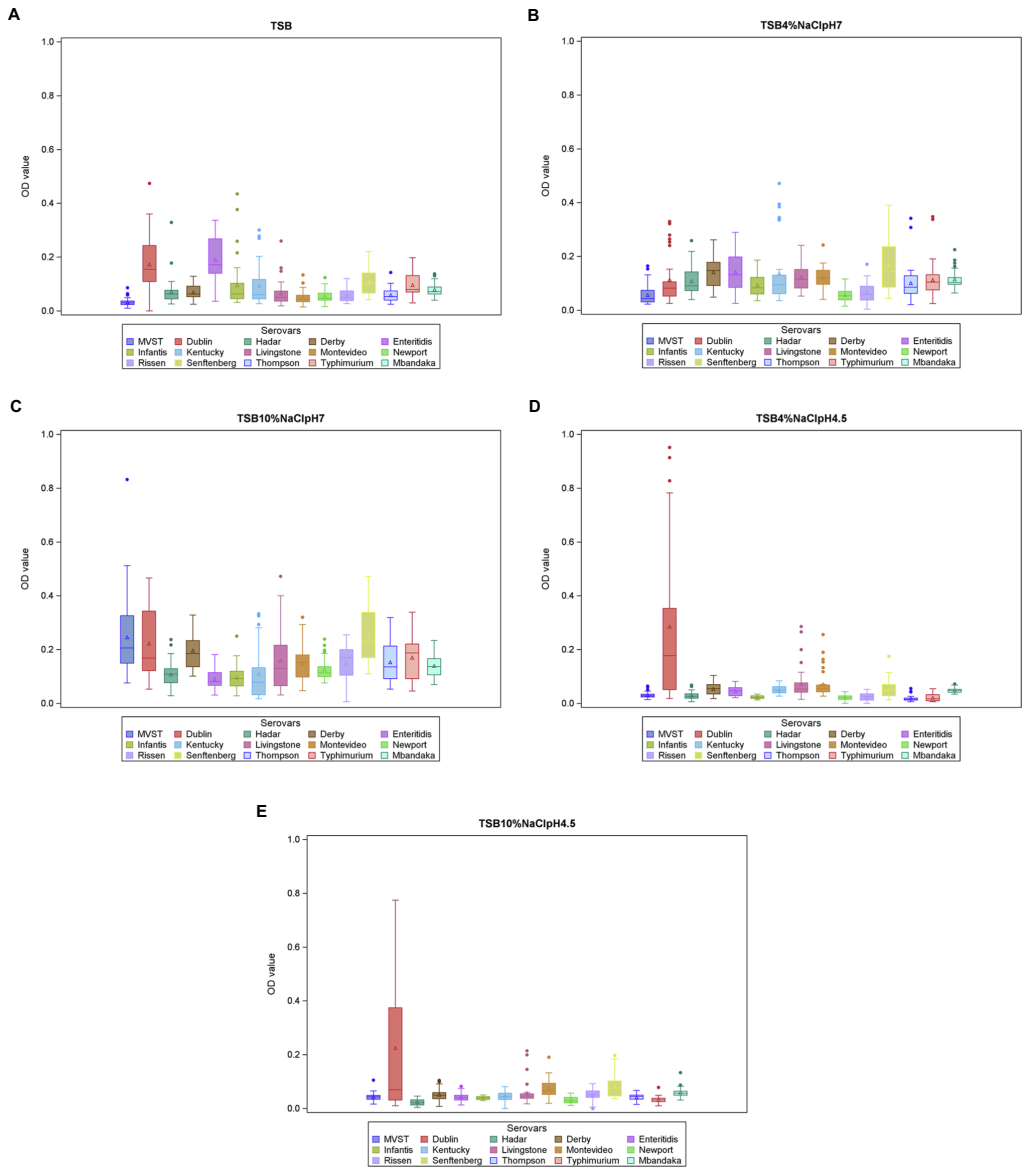
Biofilm formation (OD_570_ values) per serotype and experimental conditions (**A** = TSB, **B** = TSB pH7, 4% NaCl, **C** = TSB pH7, 10% NaCl, **D** = TSB pH4.5, 4% NaCl, **E** = TSB pH4.5, 10% NaCl). The boxes define the upper and lower quartiles and enclose the central 50% of the observations. The median is marked by a horizontal line, and the vertical whiskers extend from the box to the 2.5th percentile and the 97.5th percentile. Extreme values are indicated by dots and mean values are indicated by triangles. MVST = monophasic variant of *S*. Typhimurium.

The distribution of OD values for each experimental condition is reported in Supplementary Figure S1, while the OD value distribution for each serotype in the different conditions is summarized in Figure 1.

In the linear mixed model, the variables “serotypes,” “experimental condition,” and their interaction were significant (*p* < 0.01). The significant interaction between serotypes and experimental condition (*p* = 0.0002) suggested that the ability of each serotype to produce biofilm depends on the experimental condition. This also means that the serotypes differ in their ability to produce biofilm, depending on the experimental conditions. Focusing on the different experimental conditions, *S*. Senftenberg showed the highest OD values in TSB 10% NaCl, pH 7, and the multiple pairwise comparisons indicated that its average OD value was significantly higher than that of *S*. Infantis (Figure 1C).

*Salmonella* Dublin showed the highest OD values in both TSB 4% NaCl, pH 4.5, and TSB 10% NaCl, pH 4.5, and its average OD values at these conditions were significantly higher than those of *S*. Enteritidis, *S*. Hadar, *S*. Infantis, *S*. Kentucky, MVST, *S*. Newport, *S*. Rissen, *S*. Thompson, and *S*. Typhimurium.

In addition, *S*. Dublin average OD value in TSB 4% NaCl, pH 4.5, was also significantly higher than those of *S*. Livingstone, *S*. Mbandaka, and *S*. Senftenberg, and in TSB 10% NaCl, pH 4.5, it was significantly higher than that of *S*. Derby.

Finally, no significant differences were detected among serotypes in TSB and TSB 4% NaCl, pH 7; however, in the former, *S*. Dublin and *S*. Enteritidis produced the highest amount of biofilm (Figure 1A).

**Figure 2:**
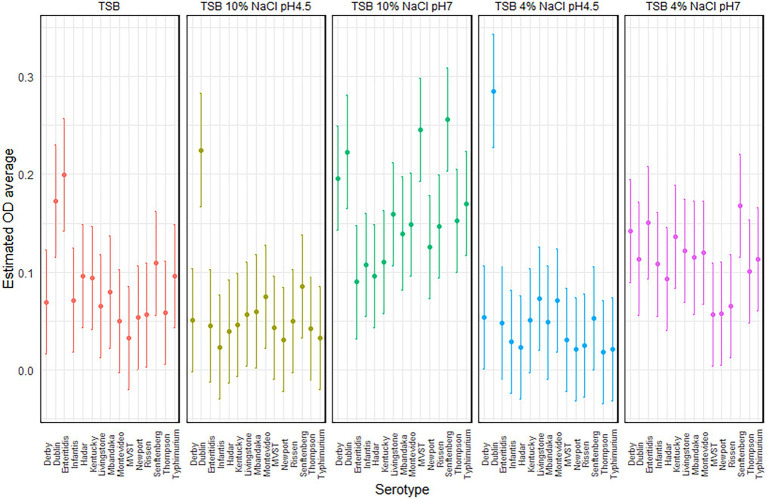
Plot of the estimated average optical density (OD) and 95% CIs, per serotype and experimental conditions.

Focusing on each serotype, MVST and *S*. Senftenberg showed a different behavior according to the experimental condition. MVST produced a significantly higher amount of biofilm in TSB 10% NaCl, pH 7, than in the other experimental condition tested, whereas *S*. Senftenberg produced a significantly higher amount of biofilm in TSB 10% NaCl, pH 7, than in TSB 4% NaCl, pH 4.5, and TSB 10% NaCl, pH 4.5 (Figure 2).

The significant interaction between serotypes and experimental condition (*p* = 0.0002) suggests that the ability of each serotypes to produce biofilm depended on the experimental conditions; this means also that the serotypes have a different capacity to form biofilm, both between serotypes and within a serotype, that depends on the experimental conditions.

## Discussion

The ability of different bacteria to form biofilm has long been considered a key factor for survival and persistence in different environments (Hall-Stoodley et al., 2004; Bjarnsholt et al., 2018). Different factors, including pH, temperature, and incubation period, influence the biofilm formation process (Stepanović et al., 2004; Agarwal et al., 2011; Díez-García et al., 2012; O’Leary et al., 2015; Roy et al., 2021). *Salmonella* strains are able to form biofilms on different abiotic surfaces, including polystyrene microplates (Stepanović et al., 2004; Steenackers et al., 2012), and it especially forms biofilm under nutrient-deficient conditions (Hood and Zottola, 1997; Ngwai et al., 2006; Shatila et al., 2021), since the promotor responsible for biofilm formation (*agfD*) reaches maximum expression levels in starvation conditions (Gerstel and Römling, 2001). Moreover, under limited nutrient conditions, bacterial surface characteristics, such as hydrophobicity and irreversible attachment, are altered, and close association with the surface is more efficient (Hood and Zottola, 1997; Ngwai et al., 2006). In the current study, we studied the ability of *Salmonella* strains belonging to different serotypes to form biofilms on microtiter plates under different NaCl and pH conditions, using TSB as medium. TSB is considered less favorable for *Salmonella* growth than other laboratory media, and it has been previously used in other studies to test the ability of *Salmonella* to form biofilm (Stepanović et al., 2004; Lianou and Koutsoumanis, 2012). We varied pH and salinity to reflect environmental conditions of food processing environments, as this has been shown to significantly affect biofilm production (Stepanović et al., 2004; Lianou and Koutsoumanis, 2011). As the most extreme conditions, we chose pH 4.5, as according to Bell and Kyriakides (2009) most *Salmonella* strains are not able to grow below pH 4.5, and NaCl concentrations of 4 and 10%, in order to obtain TSB with a_w_ 0.976 and 0.94, respectively. We assessed the ability to form biofilm for a selection of 88 *Salmonella* strains belonging to 15 different serotypes, including serotypes that are most frequently isolated from human infections.

Considering the overall tested conditions, only a limited number of isolates (*n* = 14) were not able to produce biofilms, indicating that biofilm formation is a process activated by most *Salmonella* bacteria to cope with stressful environmental conditions (Spector and Kenyon, 2012). The highest number of isolates classified as no biofilm producers was recorded in TSB 4% NaCl, pH 4.5, probably because this condition is a limit for growth of most serotypes. Contrary to Römling et al. (1998), who observed inhibitory effect of high concentrations of NaCl on *agfD* expression; the tested strains in the current study were classified as moderate and strong biofilm producers in TSB 10% NaCl, pH 7, in 68.2 and 11.4% of the cases, respectively. The same strains, when tested in TSB 10% NaCl, pH 4.5, however, did not show the same ability to produce biofilm, and the great majority of isolates was classified as weak producers (90.9%). This combination of high salt and low pH seems to hamper the ability of strain to form biofilm, as already reported also by others (Lianou and Koutsoumanis, 2012; Moraes et al., 2018; Roy et al., 2021).

The results also suggested that pH overall had a stronger effect on biofilm production than osmolarity. Indeed, we noted a general reduction in the ability to form biofilm among isolates of *Salmonella* at pH 4.5. Previous studies have reported that biofilm formation increased at increasing pH values, with optimal condition at neutral pH (Lianou and Koutsoumanis, 2012; Iliadis et al., 2018). In concordance with this, we observed the highest number of strains classified as moderate and strong biofilm producers when they grew on TSB at pH 7.

In agreement with previous studies (Stepanović et al., 2004; Agarwal et al., 2011; Lianou and Koutsoumanis, 2012), differences in biofilm production were observed among serotypes when *Salmonella* strains formed biofilms on plastic surfaces; however, the ranking of serotypes was not consistent throughout all the growth conditions evaluated. Similar observations were reported by Vestby et al. (2009) and Díez-García et al. (2012), who found substantial differences among serotypes and, in particular, they highlighted the great ability of *S*. Agona to form biofilm, compared to other serotypes. Of note, both studies reported variable capacity to produce biofilm by *S*. Typhimurium, with strains belonging to that serotype being classified as weak, moderate, or strong (Vestby et al., 2009; Díez-García et al., 2012). We observed within serotype variability in the biofilm-forming activity for *S*. Typhimurium. However, *S*. Typhimurium strains were classified as “weak” or “moderate,” with only one isolate being “no biofilm producer” in TSB 4% NaCl, pH 4.5, and one isolate being “strong biofilm producer” in TSB 10% NaCl, pH 7. Other serotypes which showed high variability in their ability to form biofilm were *S*. Derby, *S*. Infantis, and *S*. Enteritidis, as described also by Díez-García et al. (2012) and Lianou and Koutsoumanis (2012). A high variation in biofilm-forming behavior is not only a characteristics of *Salmonella*, but has also been reported for other food relevant pathogens, such as *Listeria monocytogenes* and *Escherichia coli* (Borucki et al., 2003; Reisner et al., 2006; Nilsson et al., 2011; Schiebel et al., 2017).

From our data, *S*. Dublin emerged as the serotype with the greatest ability to form biofilm in several conditions, namely, TSB, and TSB at pH 4.5 with both 4 and 10% NaCl. *Salmonella* Dublin is a serotype specifically adapted to cattle, but which also cause severe human infections, with high mortality rates (Funke et al., 2017; Harvey et al., 2017; Ju et al., 2018; Ung et al., 2019). In a study by Martinez-Sanguiné et al. (2021), the authors compared *in vitro* resistance to acid stress in *S*. Dublin and *S*. Enteritidis and concluded that isolates of *S*. Dublin were more resistant to the stress conditions found during infection, contributing to its higher invasiveness compared to *S*. Enteritidis. In this sense, the requirement of an acid resistance response to survive the harsh acidic conditions of the stomach or inside the *Salmonella*-containing vacuole once the bacteria become intracellular has been reported and could contribute to the higher invasiveness of this serotype compared to others. The ability to form an extracellular matrix at pH 4.5 could contribute to the survival of *S*. Dublin isolates in such acidic environments, since aggregation could represent a mechanism to ensure that a sufficient, even though low, inoculum reaches the epithelial layer and cause infection (Collinson et al., 1991). With regard to the ability of *S*. Dublin to form biofilm even at high salt concentration, our results disagree with those by Ju et al. (2018), who cultured *S*. Dublin strains in glucose-free medium with different NaCl concentrations (0–2% w/v) and observed that NaCl seems to be indispensable to form biofilm, but has adverse effects at high concentration. The reason for the discrepancy is unknown, but could be related to the use of different growth media.

Serotypes frequently isolated from human infections, such as *S*. Typhimurium and its monophasic variant and *S*. Enteritidis, did not show great biofilm-formation abilities at pH 4.5. In particular, *S*. Typhimurium strains were classified as weak or moderate biofilm producers in the tested conditions. The observations agreed to those by O’Leary et al. (2015), who investigated the biofilm-forming ability of 142 *S*. Typhimurium isolates, of which 90.8% formed weak to moderate biofilms. Interestingly, in the current study, the monophasic variant of *S*. Typhimurium, a serotype frequently isolated from humans, significantly differed in its ability to form biofilm between the experimental conditions. This behavior was observed also for *S*. Senftenberg, which showed significantly more biofilm formation than the rest of the serotypes in TSB supplemented with 10% NaCl. Although this serotype is rarely isolated from humans (only 119 reported cases in the EU in 2019, ECDC, n.d.), recently the number of isolations from animals, especially in the poultry sector, and ready-to-eat vegetables (fresh basil) arose (Pezzoli et al., 2007; Boumart et al., 2012). The concern with this serotype is derived from its high resistance to antimicrobials (Hendriksen et al., 2013; Veeraraghavan et al., 2019) and persistence in food processing environments (Boumart et al., 2012; Grépinet et al., 2012; El Ghany et al., 2016). As already reported by Vestby et al. (2009), the ability of *S*. Senftenberg to form biofilm could be accounted as an important factor for persistence, especially in the food processing environments, posing a great risk for food contamination and spoilage.

With respect to the other serotypes studied, we did not observe trends linked to the serotype in biofilm-formation ability, and this seems to be a general trends also reported by other researchers when screening a large number of *Salmonella* isolates, belonging to multiple serotypes (Agarwal et al., 2011; Lianou and Koutsoumanis, 2012; Moraes et al., 2018). Traditionally, bacterial foodborne pathogens are regarded as pathogenic at the species level; nonetheless, there is increasing scientific evidence demonstrating a subtype-dependent virulence potential, supported by a corresponding variation in various aspects of microbial behavior, including biofilm formation (Lianou and Koutsoumanis, 2013; Lianou et al., 2020). Moreover, we noticed substantial variability in the biofilm-forming behavior of the tested strains, with differences both intra-serotype and intra-conditions. Different papers already reported high inter-isolate variability of the biofilm-formation behavior, with regard to incubation media and time (Díez-García et al., 2012; Castelijn et al., 2013; Lianou et al., 2020). The source of *Salmonella* isolates did not seem to influence the biofilm formation ability on plastic surfaces, as reported by other researchers (Stepanović et al., 2004). This was not surprising, since ultimately, human isolates originate in food, which has been contaminated by *Salmonella* from livestock.

In conclusion, despite the limited number of strains tested for each serotype, our data suggest that the capacity of *Salmonella* strains to form biofilm might be strain- and experimental condition-dependent, as reported also for other foodborne pathogens, such as *Bacillus cereus*, *Staphylococcus aureus*, *Campylobacter jejuni*, and *E. coli* (Lianou et al., 2020). It is thus advisable to consider the ability of *Salmonella* at a strains level, rather than at a serotype level, to form biofilm as one of the events contributing to the persistence in the food processing environments and products, serving as a reservoir also for human infections.

Although the literature is not conclusive about the possibility for the strains able to form biofilms to be also more virulent or responsible for outbreaks (Aviles et al., 2013; Etter et al., 2019; Romeu et al., 2020), and despite some authors suggesting that host adaptation in *Salmonella* generally causes a reduction in the ability to form biofilm (Römling et al., 2003; MacKenzie et al., 2017), we noted that *S*. Dublin was the best serotype under several condition. The serotypes most commonly causing human infections in Europe were not superior in biofilm formation, suggesting that this phenotype is not decisive in the overall success of serotypes in terms of human infections.

## Data Availability Statement

The original contributions presented in the study are included in the article/Supplementary Material, further inquiries can be directed to the corresponding author.

## Author Contributions

SP conceived the work, performed the experiments, and wrote the manuscript. SD performed the experiments. MM performed the statistical analyses. JO, CL, and LB critically reviewed the manuscript. CL and LB contributed to the concept of the work. All authors contributed to the article and approved the submitted version.

## Funding

This work was supported by the project “PRoSPECT: Predicting Salmonella Pathogenic Potential to Enhance Targeted Control Strategies,” funded by the Italian Ministry of Health (Grant No. RF-2018-12366604).

## Conflict of Interest

The authors declare that the research was conducted in the absence of any commercial or financial relationships that could be construed as a potential conflict of interest.

## Publisher’s Note

All claims expressed in this article are solely those of the authors and do not necessarily represent those of their affiliated organizations, or those of the publisher, the editors and the reviewers. Any product that may be evaluated in this article, or claim that may be made by its manufacturer, is not guaranteed or endorsed by the publisher.
