# Supplementary material for: Effect of pH and Salinity on the Ability of Salmonella Serotypes to Form Biofilm
Source: Front Microbiol. 2022 Apr 7;13:821679. doi: 10.3389/fmicb.2022.821679 (PMC9021792; doi:10.3389/fmicb.2022.821679)
Supplement: Supplementary file 1 [file Data_Sheet_1.docx]

**Supplementary Material and Method**

**Model specification**

The model used in this analysis is:

$$y_{ijkz}=x_{ijkz}\beta+u_{k/ij}+\varepsilon_{ijkz}$$

where y_ijkz_ represents the OD-value of dependent variable for the replicate *z* of the isolate *k* nested within the serovar *j* and medium *i*. β represent the vector of fixed intercept and fixed effects (serovars, medium and their interaction); *u_k/ij_* is the random effect associated with the intercept for isolate *k* within serovar *j* in medium *i*; and ε*_ijkz_* represents the residual.

The distribution of the random effects associated with isolate nested within a given serovar in medium is

$$u_{k/ij}\sim N(0,\sigma_{int: isol}^{2})$$

where $\sigma_{(int:isol)}^{2}$ represents the variance of the random isolate-specific intercepts at any given serovar in medium. This between- isolate variance is assumed to be constant for all serovars in medium. The distribution of the residuals associated with the replicates-level observations is

$$\varepsilon_{ijk}\text{\textasciitilde N(0, }\sigma^{2})$$

where σ^2^ represents the residual variance.

Random effects *u_j|k_*, and ε*_ijk_* are assumed all mutually independent.

**Supplementary Results**


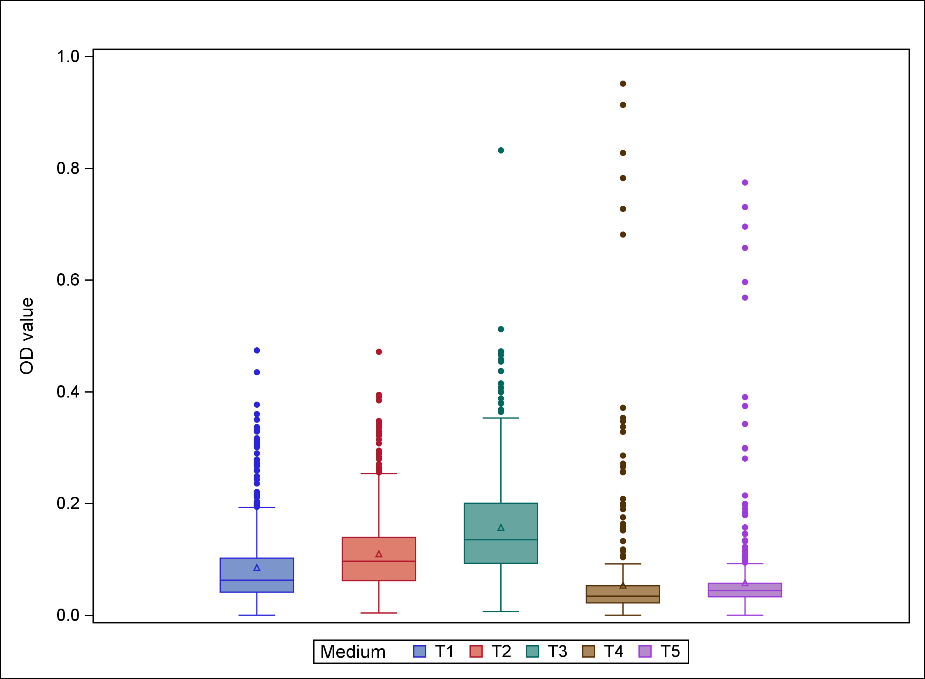


**Supplementary Figure S1**. OD values distribution per experimental condition (T1=TSB, T2=TSB pH 7, 4% NaCl, T3= TSB pH 7, 10% NaCl, T4= TSB pH 4.5, 4% NaCl , T5= TSB pH 4.5, 10% NaCl)
